# New Appliances and Things Medical

**Published:** 1902-01-18

**Authors:** 


### NEW APPLIANCES AND THINGS MEDICAL.

**[We shall be glad to receive, at our Office, 28 & 29 Southampton Street, Strand, London, W.C., from the manufacturers, specimens of all new preparations and appliances which may be brought out from time to time.]**

### ANTIFIEVRES ROBERT.

(London Agent: Gilbert, Kimpton and Co., 19 St. Dunstan's Hill, London, E.C.)

This preparation is claimed to be a radical cure for agues, intermittent and other marsh fevers. Although we must accept with reserve any statements which claim complete cures for these diseases by medicinal means, Robert's Anti-Pyretic appears to have a very decided influence in cutting short or terminating an attack.

### THE ODOURLESS RETENTIVE DISINFECTING CLOTH.

(The Odourless Retentive Disinfecting Cloth Company, 96 Leadenhall Street, London, E.C.)

This new disinfecting cloth is intended to be used for wiping surfaces and objects which are liable to become infected with disease germs and which may, consequently, be sources of danger to the public. The seats of water-closets have long been regarded as possible media for conveying disease, and within the last few months attention has been called to the dangers arising from a number of persons, some of them probably suffering from infectious complaints, speaking into the same telephone receiver. A disinfecting cloth, such as that before us, has obvious advantages in the removal of such sources of infection. It is intended to be kept permanently at hand in places in which its services may be of use. The cloth itself remains moist almost indefinitely, owing to the hydroscopic character of the substance with which it is impregnated.

### PINE FIBRE.

(Davis's Feather Mills, Limited, 61-63 High Street, Whitechapel, E.)

Pine Fibre may be used for the purposes of upholstery in which horsehair is usually employed. It has practically the same properties, that is to say it is springy and resilient, and has good lasting qualities; at the same time it is cool, cleanly and but slightly absorbent. It is, moreover, an inexpensive substitute for horsehair, which renders it particularly useful for the stuffing of pillows, mattresses, etc., in hospitals and institutions. The fibre is prepared by a special process from the growth of the long-leaf pine of the Southern States of America, and contains in some degree the healthy odour which is natural to the pine, but pleasant to the sense of smell. As is well known vermin and insects avoid as far as possible the neighbourhood of pine forests, or substances which are impregnated with the turpentines, camphors or resins derived from the wood-bark or leaves of the trees. The repellent action of pine fibre with regard to vermin will be appreciated under circumstances in which pests of this kind are suspected.

